# Enhanced attraction between drops carrying fluctuating charge distributions

**DOI:** 10.1098/rspa.2021.0714

**Published:** 2022-01

**Authors:** M. H. P. Ambaum, T. Auerswald, R. Eaves, R. G. Harrison

**Affiliations:** ^1^ Department of Meteorology, University of Reading, Reading, UK; ^2^ Department of Atmospheric, Oceanic and Planetary Physics, University of Oxford, Oxford, UK

**Keywords:** drops, electrostatics, rain formation

## Abstract

The electrostatic force between conductive spheres is always attractive at small separations, irrespective of their mean charge, when the charge on the spheres is constant. In many situations, the charge may not be fixed, such as for water drops in the natural atmosphere which vary in size and charge. We show that the attractive force between charged conductive spheres increases with increasing charge variance. The importance of this unrecognized electrostatic effect between water drops is evaluated for its potential to enhance rain formation.

## 1. Introduction

Air has long been recognized to contain aerosol particles and droplets which can become electrically charged, such as in fogs [1] or dusts [2]. Droplet charging arises primarily from exchange of charge with the cluster ions continuously formed by radioactivity and cosmic rays (e.g. [3]). These ions are highly mobile. Chemical differences between positive and negative cluster ions cause a natural asymmetry in their diffusive properties [4]. This asymmetry leads to a non-zero charge on the drops to which the ions diffuse. The effects of charge on the behaviour of drops and many aerosols (from now on simply referred to as ‘drops’) can be investigated by modelling them electrically as conducting spheres.

In this paper, we will show that any variability in charge across the drops leads to a net mean attraction between the drops, irrespective of the mean polarity of the charge of the drops. As for London–van der Waals dispersion forces, these attractive forces are relatively short range, but will dominate over any repulsive forces at close enough range.

This has implications for our understanding of the role of electrical effects in processes such as warm rain formation or aerosol coagulation.

Treating cloud drops as conducting spheres is a good approximation to reality and it simplifies the problem of their electrostatic interaction considerably when compared with the interaction between dielectric spheres [5]. Firstly, cloud drops, particularly in warm clouds, can only form when there is some solute in them (usually ionic in nature, because of the strong polarity of water molecules), which through the Raoult effect suppresses the required supersaturation for drop formation [6]. This gives those cloud drops a finite conductivity, and for the zero-frequency effects we are studying in this paper, drops effectively behave as a conducting sphere. A second important factor, particularly for drops of purer water, is the high dielectric constant (around 80) of water, which essentially forces the electric field inside the sphere to become divergence free and the static electric field becomes equivalent to that in a conducting sphere [7].

The electrostatic force between two conducting spheres is in fact a remarkably involved problem [8,9]. Of course, as long as the distance between the drops is substantially larger than the radius of either drop, then the electrostatic force is given by the inverse square Coulomb law, for charges considered at the centre of the drops. However, for drops of a conducting or highly polarizable substance such as water in which charges can migrate, the detailed situation is more complicated, and the charges in one drop will induce image charges in the other drop. Those induced image charges will induce further image charges in the original drop, and then again in the second drop, repeating indefinitely, which leads to an attractive force which can dominate if the drops are close to each other.

Calculation of the force will involve the placement of an infinite, convergent series of mutually induced image charges, followed by the summation of the forces between all the image charge pairs. We will demonstrate the detail of this procedure in the next section. Recently, this infinite series has been shown to be expressible in a closed form [10], which nonetheless in practice will also involve a series expansion requiring numerical evaluation.

These force calculations all rely on the charge on the drop being a fixed quantity. In reality, the charge on a drop is a variable quantity determined by a thermal equilibrium for diffusion of ions near a charged sphere, sometimes with the added influence of ion migration driven by electric fields.

In the simplest case of a negligible external electric field, typical for fair weather conditions when layer clouds are present, the time scale for diffusion of ions to drops of radius a is proportional to 1/aZ, where Z is the drop number per unit volume [11]. This time scale is typically about 100 s, for 10 μm radius droplets with $Z=100\;{\text{cm}}^{-3}$. When an electric field is present, leading to ordered motion of the ions, the charging time can be more rapid [12]. This shows that drops interacting in a natural environment will exhibit a varying charge, which is more closely modelled as a stochastic variable rather than a fixed quantity.

In the next section, the theoretical development is laid out on how replacing a fixed charge by a stochastic variable changes the interaction force between two drops. In §3, the stochastic nature of the drop charge is discussed, and consequences for actual calculated forces between drops are demonstrated. In §4, we discuss how our results may alter rain formation processes, including modified collision kernels as well as simulations of expected effects on warm rain formation.

## 2. Theoretical development

**Figure 1.**
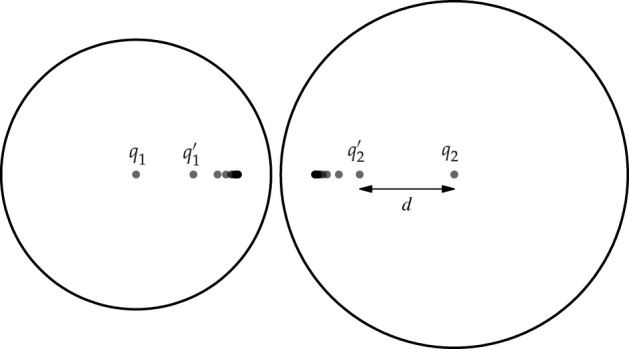
Location of image charges in two spherical drops close to each other. Each image charge induces a new image charge in the other drop, leading to an infinite series of mutually induced image charges. Note that the location of the image charges converges rapidly to a fixed point.

When two drops of radii $a_{1}$ and $a_{2}$ and charges $q_{1}$ and $q_{2}$ are placed close to each other, the charges will induce image charges in the other drop so as to ensure that the drop surface is a surface of constant potential. The image charge induced by charge $q_{1}$ (figure 1) will induce an image charge $q_{2}^{\prime }$ in the second drop at an offset $d_{2}^{\prime }$ from the drop centre, where

2.1 $$q_{2}^{\prime }=-q_{1}\frac{a_{2}}{r}\;\text{and}\;d_{2}^{\prime }=\frac{a_{2}^{2}}{r}.$$

Here, r is the distance between centres of the drops. Equivalently, the charge $q_{2}$ of the second drop will induce an image charge $q_{1}^{\prime }$ in the first drop. These image charges will, in turn, induce secondary image charges in the opposite drops of $q_{1}^{\prime \prime }$ and $q_{2}^{\prime \prime }$, where the distances between the image charges and the centres of the opposite drops are to be used for the value of r in the calculations.

Charge conservation requires that the induced image charges are compensated by an adjustment of the central charge to

2.2 $$q_{1}^{\left(0\right)}=q_{1}-q_{1}^{\prime }-q_{1}^{\prime \prime }-q_{1}^{\prime \prime \prime }-\ldots =q_{1}-\sum_{n=1}^{\infty }q_{1}^{\left(n\right)},$$

and equivalently for $q_{2}^{\left(0\right)}$.

We end up with an infinite series of image charges, and adjusted central charges, which will interact via Coulomb forces, pairwise across the drops. The total force between the two drops is therefore

2.3 $$4\pi \epsilon F=\sum_{m,n=0}^{\infty }\frac{q_{1}^{\left(m\right)}q_{2}^{\left(n\right)}}{{\left(r-d_{1}^{\left(m\right)}-d_{2}^{\left(n\right)}\right)}^{2}},$$

with ϵ the relevant permittivity of the medium in which the drops reside, and $d_{1}^{\left(m\right)}$ and $d_{2}^{\left(n\right)}$ the displacements from the drop centres for image charges $q_{1}^{\left(m\right)}$ and $q_{2}^{\left(n\right)}$, respectively. A repulsive force corresponds to a positive value for F. We will only consider interactions between two drops where the force is always aligned with the difference vector between the centres of the drops. This means that all calculations only involve scalar quantities.

An effective approximation of the total force, which gives good results for a broad range of parameters, includes the standard Coulomb force between $q_{1}$ and $q_{2}$ plus the attractive force between $q_{1}$ and the induced finite size dipole $q_{2}^{\prime }d_{2}^{\prime }$ plus the attractive force between $q_{2}$ and the induced finite size dipole $q_{1}^{\prime }d_{1}^{\prime }$, an approximation similar to the one employed in [13]. This approximation leads to a force equal to

2.4 $$4\pi \epsilon F=\frac{q_{1}q_{2}}{r^{2}}-q_{1}^{2}\frac{a_{2}}{r}\left(\frac{1}{{\left(r-a_{2}^{2}/r\right)}^{2}}-\frac{1}{r^{2}}\right)-q_{2}^{2}\frac{a_{1}}{r}\left(\frac{1}{{\left(r-a_{1}^{2}/r\right)}^{2}}-\frac{1}{r^{2}}\right).$$

The key outcome is that the force, even at higher-order approximations, can be written as the sum of three components, respectively, proportional to $q_{1}q_{2}$, $-q_{1}^{2}$, and $-q_{2}^{2}$. The last two components are always attractive, and can dominate at close enough distance, except in certain symmetric cases where the attraction only occurs when the drops touch. The sign of the zeroth-order contribution of course depends on the signs of $q_{1}$ and $q_{2}$, and is repulsive for like-signed charges.

We will next assume that the charge on each drop is in fact a stochastic variable, set by a thermal equilibrium for the flux of environmental ions of either polarity to the drops. In the next section, we will discuss the physical basis for such an assumption. For now we will assume that the charges are independent stochastic variables with means ${\bar{q}}_{1}$ and ${\bar{q}}_{2}$ and standard deviations $\sigma_{1}$ and $\sigma_{2}$.

We can see that, under these stochastic assumptions, the Coulomb force (the first term in equation (2.4)) is not changed owing to the two stochastic charges being independent. However, the magnitude of the attractive forces (the second and third terms in equation (2.4)) is increased: the term $q_{1}q_{2}$ is replaced by ${\bar{q}}_{1}{\bar{q}}_{2}$, while the terms $q_{1}^{2}$ and $q_{2}^{2}$ are replaced by ${\bar{q}}_{1}^{2}+\sigma_{1}^{2}$ and ${\bar{q}}_{2}^{2}+\sigma_{2}^{2}$, respectively.

An interesting example occurs for the stochastic situation where the mean drop charge is zero. For point charges the mean force between the drops will then vanish but for finite size drops we find an attractive force approximately equal to

2.5 $$4\pi \epsilon F=-2\frac{\sigma_{1}^{2}a_{2}^{3}+\sigma_{2}^{2}a_{1}^{3}}{r^{5}},$$

where, for simplicity, we approximated equation (2.4) for $r\gg a_{1}$, $a_{2}$. Whatever the level of approximation used, we find an attractive force proportional to the variance of the charges. The negative power of r in the attractive force is smaller, and therefore longer range, than the sixth power dependence in the London–van der Waals case, because here we consider attraction between two independent stochastic charges and their induced dipoles.

For nearby drops, it is expected that any variability in the two drop charges will be anti-correlated as ions captured by one drop cannot be captured by the other drop. In that case the modified Coulomb part, proportional to $q_{1}q_{2}$, will also contain a contribution proportional to $\text{cov}(q_{1},q_{2})$ which is negative and therefore helps the attraction between drops. It is hard to quantify how large this contribution will be.

In summary, treating the charges as stochastic variables leads to an interaction force which is augmented by a mean attractive part proportional to the variances of the charges. In the next sections, we quantify the stochastic nature of the charge for small drops and give explicit examples of the stochastic effect on the electrostatic force between the drops and the possible effects on rainfall generation in clouds.

## 3. Boltzmann equilibrium charge distribution

For the simplest case of a diffusion charging of a monodisperse aerosol of radius *a*, in the presence of equal concentrations of positive and negative ions having the same mobility, the fraction of the particles carrying j elementary charges, $N_{j}$, is found experimentally (e.g. for oil drops [14]) to be given by

3.1 $$\frac{N_{j}}{N_{0}}=\exp\left(-\frac{j^{2}e^{2}}{8\pi \epsilon akT}\right),$$

with k the Boltzmann constant and T the absolute temperature. For very small drops ($a<10^{-8}\;\text{m}$), there is some departure from this relationship [15,16]): Schrödinger pointed out that this was likely to be due to the breakdown of the continuum assumption for charge at such small sizes [17].

The basic form of equation (3.1) is typically explained [18] as arising from a Boltzmann factor $e^{(-\varepsilon )/kT}$ where the free energy ε corresponds to the energy of an isolated sphere carrying a charge Q. If the total charge Q on the sphere consists of j elementary charges e, and assuming the capacitance of an isolated sphere to be C=4πϵa, the relevant electrical energy is

3.2 $$\frac{1}{2}CV^{2}=\frac{1}{2}\frac{Q^{2}}{C}=\frac{j^{2}\;e^{2}}{8\pi \epsilon a}.$$

Consequently, equation (3.1) is sometimes known as the Boltzmann charge distribution. (Equation (3.1) can alternatively be derived by considering detailed balance [19,20] using ion–aerosol attachment coefficients [21], from which it is apparent that the Boltzmann charge distribution is the limiting case when both the bipolar ion concentrations and ion mobilities are equal—Clement & Harrison [19] argue for a modified Boltzmann distribution in the more general case.) The mean of the Boltzmann charge distribution is zero, as the distribution is symmetrical from negative to positive. A more relevant consideration is the proportion of charged drops present, because of the consequence for electrostatic effects on and between the drops. By integrating across the Boltzmann charge distribution, the fraction of charged droplets $f_{c}$ is given by

3.3 $$f_{c}=1-\sqrt{\frac{\lambda }{\pi }},$$

where $\lambda =e^{2}/8\pi \epsilon akT$. For monodisperse assemblies of droplets with radii between 0.1 μm and 20 μm, $f_{c}$ varies between about 0.7 and 0.98 at $5^{\circ }\text{C}$ (although the temperature dependence is negligible), indicating that the majority of the droplets present will be charged. If the properties differ between positive and negative ions, the fraction of the droplets charged for any monodisperse droplet size will be even greater than that given by equation (3.3) (e.g. see the asymmetry enhancement factor in eqn (47) of [20]).

The width of the charge distribution can be described by analogy between the (symmetrical) Boltzmann distribution and a Gaussian distribution of zero mean, i.e. a standard deviation in j of

3.4 $$\sigma_{j}=\sqrt{\frac{1}{2\lambda }}.$$

An example calculation for a drop of 10 μm radius at a temperature of $5^{\circ }\text{C}$ leads to a charge standard deviation of 13 elementary charges.

Hence, by considering either the proportion of charged droplets present or the variability in their charge, it is clear that a range of droplet charges is always expected to occur in natural conditions. If, in addition, the local ion concentrations are unequal, the asymmetry from this and their mobility differences will lead to greater charging, with a non-zero mean charge in the droplet charge distribution.

We conclude this section by showing some examples for the electrostatic force between charged drops, and the additional effects of allowing charge stochasticity.

**Figure 2.**
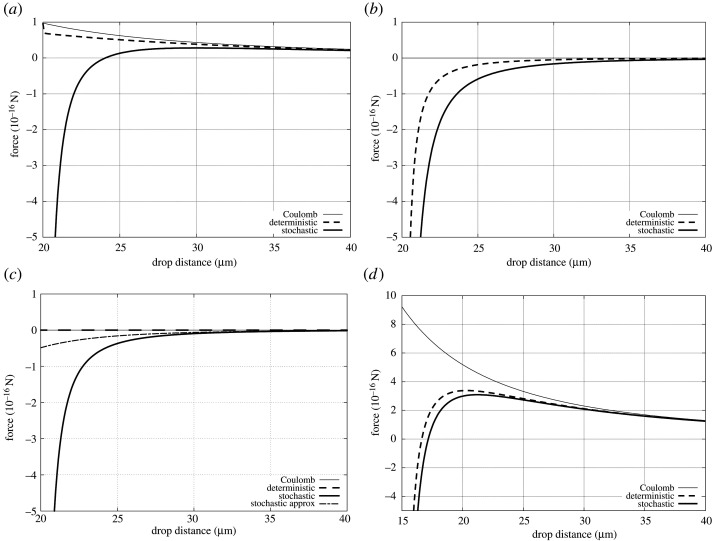
Force between drops (in $10^{-16}\;\text{N}$) as a function of the distance between the drop centres (in μm). (a) Two drops of radii 10 μm, charges 13e, standard deviations 13e. (b) Two drops of radii 10 μm, charges 0e and 13e, standard deviations 13e. (c) Two drops of radii 10 μm, charges 0e, standard deviations 13e. (d) Two drops of radii 5 μm and 10 μm, charges 30e, standard deviations 13e.

In figure 2, we collate a set of examples for forces between small charged and uncharged drops. There is no simple scaling of the force equations with charge, or distance, or drop size, so we can only attempt to give a flavour of the possible effects. The calculations of the forces with fixed charges, hereafter labelled *deterministic*, use equation (2.3). Alternatively, treating charge as a stochastic variable, we assume that the adjustment of the image charges is effectively immediate, so that we can calculate the average force (hereafter labelled *stochastic*) for two charges drawn independently from normal distributions of the given mean and standard deviation. In figure 2, we used 4096 realizations of this force to calculate its average. Either force can be compared with the Coulomb force between corresponding point charges, which is also included in figure 2. The parameter values chosen are in the figure caption.

Figure 2*a* shows the force between two equally charged drops of equal size. Both the Coulomb force and deterministic force are repulsive, although at the point where the drops touch the image forces introduce a singular attraction, which is not visible in the figure. This singularity is pathological as it occurs only for chosen symmetrical parameters.

The same figure shows the force when the charges are treated as stochastic variables. Although at large distances the repulsive Coulomb force dominates, at close range (in this case when the two drops are about 4 μm apart) the force becomes attractive. The high-order nature of the image charge effect ensures that, when the force is attractive at close range, the force rapidly attains a very large magnitude.

Figure 2*b* shows the force when only one drop is charged. The Coulomb force vanishes, as expected, but the deterministic force is always attractive, particularly at close range. The stochastic force is still more attractive with an attractive range about twice as large as in the deterministic case.

Figure 2*c* shows the force between two on average neutral drops. Both the Coulomb and deterministic forces vanish. The stochastic force is strongly attractive at close distances. For reference, the panel includes a graph of the attractive force as approximated in equation (2.5), which approximates well the stochastic force at longer ranges, but fails, as expected, at shorter ranges.

Figure 2*d* shows the force between two higher charged small drops where we may expect the Coulomb repulsion to dominate. Indeed, the repulsive force is substantially larger in this case but even here at close ranges the attractive force starts to dominate. The stochastic component, which has the same charge standard deviations as in the previous case, enhances this attraction but in this case to a lesser extent than in the deterministic case.

The absolute values of the forces remain relatively small (except at very close proximity), for example, compared with the expected gravitational force for a 10 μm radius drop. The comparison with the drop’s weight may not, however, be the most relevant for the cloud processes, as pointed out before in [22], where it was also suggested that close-range attraction could have a substantial enhancement effect on the rate at which small drops coalesce to form large rain drops (i.e. the autoconversion rate). The additional aspects introduced by considering charge as a stochastic variable suggests that this enhancement may therefore, in reality, be even greater.

## 4. Application to clouds and rain formation

Warm rain formation in the atmosphere (that is, rain formation in which frozen hydrometeors do not play a role) relies on the collision and coalescence of smaller drops to form larger drops until the drops become large enough to escape the cloudy air and fall to the ground. The principles of these processes are well understood and many textbooks on cloud physics exist which describe the details of these processes (e.g. [6,23]). Remote sensing methods can be used to observe warm cloud droplet collection processes, such as through combining reflectivity profiles from CloudSat and the Moderate Resolution Imaging Spectroradiometer (MODIS) [24].

A key variable in the formation of rain is the average rate at which two drops of different size collide, the so-called collision kernel. This kernel is set by several system parameters and variables, most prominently the drop radii.

The kernel is proportional to the drop collision cross-section, the mean relative velocity of the two drops, and is modified by hydrodynamic effects of one drop moving in the wake of the other drop. The mean relative velocity is often taken as the difference between the terminal velocities of the two drops, but in a turbulent field this is typically enhanced because of the turbulent advection of the drops [25].

When the drops are charged, the attractive force between the drops is expected to enhance the collision kernel, although the remote Coulomb repulsion between like-signed charges might actually reduce the kernel.

Here we show a single result from a model which uses an ABC flow [26] to simulate the turbulent advection and collision of drops under the influence of electrostatic interaction [27]. We express the effect of the electrostatic interactions as an enhancement factor, the ratio of the collision kernel with electrostatic effects to the kernel without electrostatic effects.

**Figure 3.**
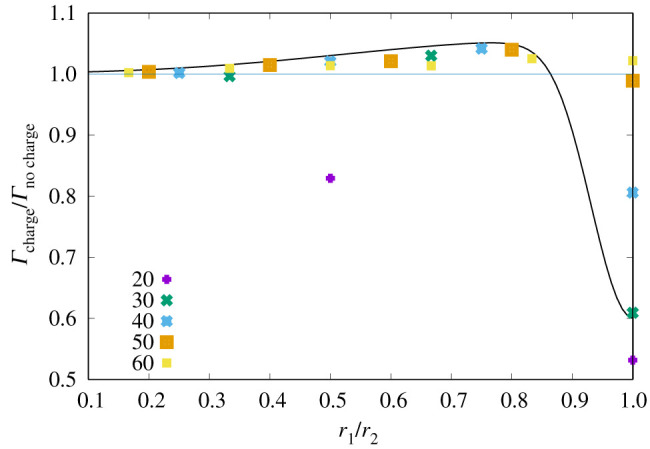
Electrostatic enhancement factor: the ratio between the collision kernels for the charged case compared with the uncharged case, as a function of drop size ratio. The keys to the symbols represent the size of the largest drop in the interaction. The black line is a smooth approximation used to modify the kernels in the charged stochastic collision equation simulation. (Online version in colour.)

Figure 3 shows one example of a case where a population of drops of sizes between 10 and 60 μm radius were simulated. The 10 μm drops had a charge of 10 000e and the charge increases linearly with the radius. This is a case where one might expect quite a strong effect of the Coulomb repulsion. The stochastic effect on the charges was simulated by using a polynomial approximation to the expected electrostatic forces.

As can be seen in figure 3, the collision kernel in this case is enhanced by a few per cent across a wide range of drop size ratios. We have found this to be the case for several parameter set-ups. For a drop size ratio close to 1, we often find a reduction in the collision kernel. This is particularly the case for smaller drops (10 μm and smaller) and highly charged drops. This can be understood by the fact that, for a drop size ratio close to 1, the relative velocity of the drops will be very small, typically too small to overcome the long-range Coulomb repulsion of like-signed charges [22]. Larger drops with their larger inertia and drag are less affected by this than smaller drops. We also observe that for small size ratios (≲0.25) the enhancement due to charge is also small. Here the natural relative velocity is already quite large in most cases and any charge effects would only moderately change the drop trajectories.

Despite all these competing effects, we find that typically the total collision rate across the drop size spectrum in this simulation is enhanced by up to 5%, although a general result is hard to obtain as this depends on the charge and size distributions considered.

A standard way to predict the evolution of a drop size spectrum over time uses the so-called *stochastic collision equation* (SCE). The SCE is a Smoluchowski equation where the collision kernels are derived from the terminal velocity of the falling drops and include the effects of the hydrodynamic wake behind the drops. The SCE uses the collision kernels to predict the rate at which drops of two sizes collide and then form a larger drop with a mass equal to the sum of the colliding drop masses. The numerical implementation used here is described in [28].

We approximated the kernel enhancement by charge by a smooth function of the ratio of the two drops, as can be seen by the black line in figure 3, and modified the kernel used in the Bott SCE model with this enhancement. We simulated a dense maritime-style cumulus cloud with an initial liquid water content of $2\;\text{g}\;{\text{m}}^{-3}$ and mean drop radius of 10 μm.

**Figure 4.**
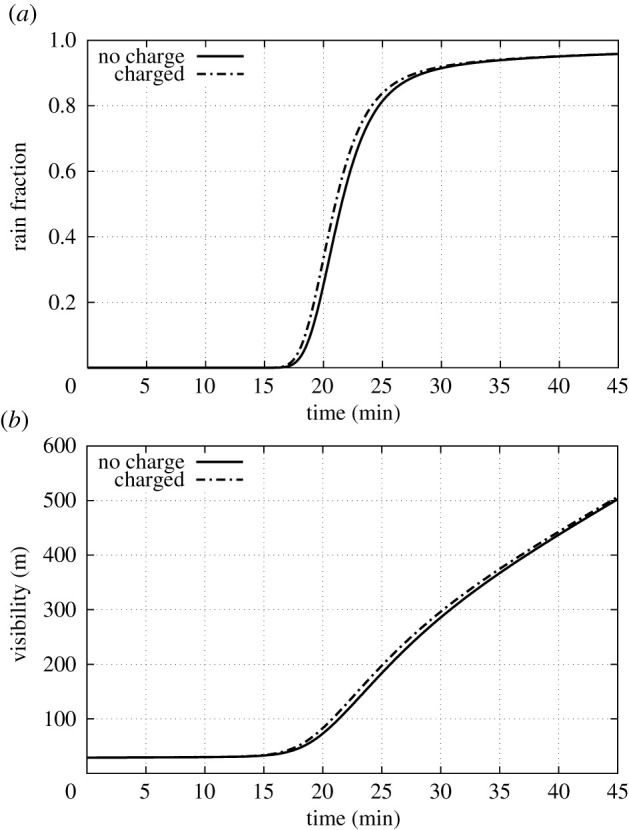
Results from simulations of the stochastic collision equation (SCE). Evolution of the rain fraction (fraction of liquid water content in drops of radius greater than 500 μm) and in-cloud visibility (using Koschmieder theory and a minimum visible contrast of 2%) for a control run and a run with collisions modified by charge.

As can be seen in figure 4, this cloud drop population evolves rapidly, with after 25 min the majority of the small drops having coagulated into drops of radius larger than 500 μm. Those drops continue to grow in size. Figure 4*b* shows the visibility, as calculated from the drop size distribution. This shows how the visibility keeps improving over time after the initial cloud drops have largely coagulated. The continued visibility increase corresponds to the continued removal of smaller drops from the drop size distribution.

The dashed curves in figure 4 show that the charging indeed speeds up the evolution of the cloud drop distribution. Despite the very modest assumed enhancement due to charge (a maximum of 5% at drop size ratios around 0.75, consistent with figure 3), competing effects at small drop size ratios and a reduction of the kernels at size ratios near 1, we find that there is a modest net speed up of the evolution of the drop size distribution, of the order of a minute or so, in fact consistent with a 5% increase in the collision rate. This indicates that the fast evolution of the drop spectrum near the 20 min mark is dominated by collisions of drops with size ratios around 0.75.

## 5. Conclusion

The electrostatic interaction between two charged spheres is a remarkably involved problem requiring the summation over an infinite series of pairs of image charge interactions. The net interaction is always attractive at short ranges, irrespective of the mean charge on the spheres.

Generally, for atmospheric situations, the charge on a drop has been considered as fixed, like that of an isolated charged sphere. However, it is more natural to consider the charge on a drop to be a stochastic variable, with fluctuations which follow from the diffusive transport of environmental ions towards the drops.

One significant consequence of this improved representation for charge as a stochastic variable is that the drop–drop attractive force is always enhanced, and acts over a longer range. The outcome for drops in the atmosphere is that electrostatic attractions between them will be larger than previously thought, and will accordingly play a different role in rain formation processes.

Applying these findings directly to warm clouds, modelling shows that collision rates are indeed enhanced by electrostatic interactions. This is despite the fact that there are several competing effects. One of these effects is that, for similar-sized drops, the relative speed of nearby drops is very similar, giving the Coulomb repulsion for like-signed drop charges the opportunity to keep drops apart. This is especially the case for smaller drops. If the drops are, on average, neutral, this Coulomb repulsion would not occur, and the stochastic attraction would in fact help the collision of the drops. Modelling the drop size distribution over time using the SCE, we find that the attractive electrostatic force dominates the evolution of the drop size distribution.

In summary, the ubiquitous presence of ion capture by drops in the atmosphere ensures that drops are attracted to each other at close ranges, irrespective of the presence of any mean drop charge. This is because the mean effect of drop charge fluctuations always yields an attractive interaction. The time scales for the evolution from cloud droplets to raindrops consequently become more rapid than for the neutral case.

## Data accessibility

The description and verification of the used three-dimensional DNS model is published as Auerswald & Ambaum [27]. The SCE model was described in Bott [28].
